# Training augmentation using additive sensory noise in a lunar rover navigation task

**DOI:** 10.3389/fnins.2023.1180314

**Published:** 2023-06-23

**Authors:** Sage O. Sherman, Anna Jonsen, Quinlan Lewis, Michael Schlittenhart, Daniel Szafir, Torin K. Clark, Allison P. Anderson

**Affiliations:** ^1^Ann & H.J. Smead Department of Aerospace Engineering Sciences, The University of Colorado Boulder, Boulder, CO, United States; ^2^Department of Computer Science, The University of North Carolina at Chapel Hill, Chapel Hill, NC, United States

**Keywords:** stochastic resonance, auditory white noise, noisy galvanic vestibular stimulation, macrocognition, long duration exploration mission, longitudinal effects

## Abstract

**Background:**

The uncertain environments of future space missions means that astronauts will need to acquire new skills rapidly; thus, a non-invasive method to enhance learning of complex tasks is desirable. Stochastic resonance (SR) is a phenomenon where adding noise improves the throughput of a weak signal. SR has been shown to improve perception and cognitive performance in certain individuals. However, the learning of operational tasks and behavioral health effects of repeated noise exposure aimed to elicit SR are unknown.

**Objective:**

We evaluated the long-term impacts and acceptability of repeated auditory white noise (AWN) and/or noisy galvanic vestibular stimulation (nGVS) on operational learning and behavioral health.

**Methods:**

Subjects (*n* = 24) participated in a time longitudinal experiment to access learning and behavioral health. Subjects were assigned to one of our four treatments: sham, AWN (55 dB SPL), nGVS (0.5 mA), and their combination to create a multi-modal SR (MMSR) condition. To assess the effects of additive noise on learning, these treatments were administered continuously during a lunar rover simulation in virtual reality. To assess behavioral health, subjects completed daily, subjective questionnaires related to their mood, sleep, stress, and their perceived acceptance of noise stimulation.

**Results:**

We found that subjects learned the lunar rover task over time, as shown by significantly lower power required for the rover to complete traverses (*p* < 0.005) and increased object identification accuracy in the environment (*p* = 0.05), but this was not influenced by additive SR noise (*p* = 0.58). We found no influence of noise on mood or stress following stimulation (*p* > 0.09). We found marginally significant longitudinal effects of noise on behavioral health (*p* = 0.06) as measured by strain and sleep. We found slight differences in stimulation acceptability between treatment groups, and notably nGVS was found to be more distracting than sham (*p* = 0.006).

**Conclusion:**

Our results suggest that repeatedly administering sensory noise does not improve long-term operational learning performance or affect behavioral health. We also find that repetitive noise administration is acceptable in this context. While additive noise does not improve performance in this paradigm, if it were used for other contexts, it appears acceptable without negative longitudinal effects.

## Highlights

- Applying vestibular and/or auditory white noise during repeated learning sessions does not affect operational task performance.
- Repetitive noise administration does not affect immediate or longitudinal behavioral health.
- Repetitive noise administration is perceived to be acceptable by users.

## Introduction

Astronauts must complete a large variety of complex, operational tasks in quick succession to accomplish mission goals; thus, they have to start training for these tasks upon being selected up until their mission. However, this training can be time consuming and many simulated environments fail to replicate the space environment accurately, as future space missions will impose unknowns that could necessitate learning new skills that were not included in mission design (Anglin et al., 2017). Skill decay is a concern for long-duration space missions as well (Pieters and Zaal, 2019). This creates the need for enhanced training techniques for on-ground and in-flight environments enabling quick skill acquisition. Additionally, the spaceflight environment creates physiological and psychological stressors that may impact cognitive and behavioral health, as well as the operator’s ability to learn (Morphew, 2020; Roy-O’Reilly et al., 2021). A technique or countermeasure aimed at enhancing training should be robust to these stressors and not further burden the crew. It is theorized that improved performance in cognitive tasks can impact learning ability (Ackerman et al., 1995; Shi and Qu, 2022). A potential training technique focused on enhancing cognitive functioning is computerized cognitive training (CCT), where subjects exercise specific cognitive concepts through computerized games in hopes of training mental ability (Jaeggi et al., 2011). However, the generalizability and transfer of construct training is often invalid for separate constructs or more complex tasks (Noack et al., 2014). Thus, it would be advantageous to develop alternative technologies that enhance cognitive functioning and learning while being appropriate for the spaceflight environment.

One such technological field being explored is neuromodulation, which refers to using a stimulus to alter nerve activity (Horn and Fox, 2020), and in some cases has been shown to enhance learning. For example, transcranial direct current stimulation (tDCS) has been shown to contribute to motor learning and motor memory formation in healthy humans (Reis et al., 2008), and improve learning in tasks involving concealed object learning (Clark et al., 2012) and recognition (Manuel and Schnider, 2016). tDCS has also been shown to improve learning for operationally relevant tasks, such as complex flight simulations (Choe et al., 2016). However, there is evidence that tDCS can leave long-lasting effects on cortical excitability after stimulation has occurred (Medeiros et al., 2012). Additionally, some neuromodulation methods may have unintentional secondary effects on other mental states, such as behavioral health. Transcranial magnetic stimulation (TMS) has been shown to reduce depression states in clinical trials (Mantovani et al., 2012). While this is a beneficial behavioral health effect, it demonstrates that there are additional side effects to these neuromodulation techniques which require further exploration. Additionally, these techniques are difficult to self-administer and can require the user to be stationary during stimulation. These limitations could make these methods inappropriate for the spaceflight environment. Considering these effects and mission constraints, it is worth exploring neuromodulation alternatives that are more applicable to the dynamic spaceflight environment. One such alternative may be stochastic resonance, which is a method of neuromodulation that is induced through noisy sensory stimulation. While there are many open questions involved in applying this technique, it could be useful for spaceflight because appropriate sensory stimulation is safe, easy to administer, requires a low design budget, and can be used in dynamic situations.

Stochastic Resonance (SR) is the phenomenon where noise improves the throughput of a non-linear signal (Moss et al., 2004). It has been postulated that this phenomenon can be utilized as a neuromodulation technique for human information processing through the use of external sensory noise. Human experimentation has shown that SR is exhibited within and across sensory channels, where perceptual thresholds (the lowest intensity stimuli a person can reliably recognize) are reduced (Zeng et al., 2000; Lugo et al., 2008; Galvan-Garza, 2018; Voros et al., 2021). Additional models in human experiments have shown that noise-enhanced sensory information could be utilized by the whole central nervous system (Hidaka et al., 2000), suggesting that SR could affect higher order neuronal processing. Along this notion, sensory noise has been shown to improve elements of cognition, such as working memory (Wilkinson et al., 2008; Söderlund et al., 2010). Further, previous research conducted in our lab showed that certain individuals may show comprehensive cognitive improvement (Sherman et al., 2023a). This evidence implies that SR could be useful for improving information processing. It is postulated that being able to efficiently encode new information can help us repair or restructure our current knowledge (Chi, 2009). So, if SR can improve mental ability it might be able to improve learning ability as active cognitive processes may improve constructive and interactive processes. When it comes to learning as a result of noise, the literature suggests that noisy galvanic vestibular stimulation (nGVS) can enhance learning of challenging locomotor tasks (Putman et al., 2021) and that auditory white noise (AWN) can improve new-word learning in adults (Angwin et al., 2017). It is unclear whether learning in these two paradigms would extend to complex operational skill acquisition through procedural memory formation. This gap in the literature warrants further investigation to understand whether sensory noise can improve learning in complex tasks.

Further, no studies were identified which investigate lasting effects of sensory noise on behavioral health (e.g., mood, stress, sleep, etc.). Considering the behavioral effects that other neuromodulation techniques have on neuronal excitability and behavioral health outcomes, it would be beneficial to know whether sensory noise induces effects that are not beneficial for spaceflight operators. Thus, this work investigates the effects that repetitive administration of sensory noise has on operational learning and behavioral health. We hypothesized that compared to a sham group (i.e., wearing hardware, but presented no noise), the groups where we applied a stimulation treatment during operations would have improved learning in our operational task (i.e., lunar rover simulation). Further, we explored the hypothesis that sensory noise would impact measures of operator behavioral health, either as a benefit or a detriment.

Noise from any sensory modality may improve signal detection and improve cognitive abilities. However, it is prudent to consider sensory modality options that integrate well with, or possibly target other complications resulting from, living in a spaceflight environment. Living in microgravity imposes sensory challenges as a result of otolithic deprivation; however, several longitudinal studies have shown that humans reinterpret and adapt their understanding of orientation and spatial surroundings in spaceflight (Pathak et al., 2022). Thus, we wanted to apply noise to sensory modalities that have demonstrated the ability to influence the vestibular, motor, somatosensory, and visual systems as these central nervous system regions are impacted and undergo sensory reweighting in spaceflight (Roy-O’Reilly et al., 2021). Directly influencing these CNS regions, nGVS has been shown to modulate spatial memory and learning in sensorimotor performance tasks (Moore et al., 2015; Hilliard et al., 2019; Putman et al., 2021). Further, nGVS has been shown to improve postural stability and perception in vestibular and visual modalities (Wilkinson et al., 2008; Wuehr et al., 2016; Galvan-Garza, 2018). Indirectly influencing these CNS regions, AWN has been shown to improve memory encoding and learning of auditory and visual stimuli (Othman et al., 2019; Sayed Daud and Sudirman, 2023). Further, AWN may influence locomotion and perception performance in visual, tactile, and somatosensory modalities (Manjarrez et al., 2007; Lugo et al., 2008; Carey et al., 2023). These two modalities have demonstrated the ability to modulate learning and improve performance in perception and key CNS regions, lending themselves as ideal neuromodulation candidates if they also improve performance in complex, operational tasks.

Thus, we chose to investigate acoustically stimulating the auditory system using AWN and electrically stimulating the vestibular system using nGVS. Additionally, these two modalities as we believed they were the least intrusive to interface design or to astronaut mobility, being ideal candidates for operating in the spaceflight environment. However, to assess this we measured the acceptability opinion of users in all groups to see whether sensory noise stimulation impacted their perceived level of ability.

## Methods

### Subjects

Twenty-four subjects (12F/12M), age 26 ± 10 years (range = 18–55 yrs) completed testing in the Bioastronautics Lab at the University of Colorado-Boulder. This research was approved by the University of Colorado-Boulder’s Institutional Review Board (protocol #21-0296) and written informed consent was obtained prior to participation. Subjects were pre-screened and excluded if they reported a history of health issues that could impact cognitive abilities, such as severe head trauma or disorders associated with thinking impairment. They were also excluded if they reported health issues that could impact auditory or vestibular processing, such as language impairment or vestibular dysfunction. Eight willing participants were excluded from being in this study for not meeting pre-screening eligibility. Additionally, subjects underwent auditory screening to verify healthy and unobstructed ear canals (via otoscopy), normal tympanometry, and normal hearing (audiometric thresholds ≤25 dB HL up to 8 kHz). No subjects that passed pre-screening eligibility were excluded as a result of this auditory screening. Twenty subjects reported their occupation as undergraduate or graduate students in technological majors and four subjects reported their occupation as engineers.

### Study design and timeline

A between-subject longitudinal experimental design was implemented to evaluate the lasting operational and behavioral health effects of repeated noise exposure. Four groups (*n* = 6 in each group, 3F/3M) were assigned a noise stimulation treatment that was used for the duration of the experiment. Subjects were assigned to treatments using a covariate randomization technique to ensure equal sex grouping in each treatment. These treatments included a no noise sham (3F/3M, age 25.3 ± 6.1 years, range = 21–34), AWN with an intensity of 55 dB SPL (3F/3M, age 27 ± 11.5 years, range = 20–50), nGVS with an intensity of 0.5 mA (3F/3M, age 22.2 ± 5.1 years, range = 18–32), and the combination of the AWN and nGVS treatments, termed multi-modal SR (MMSR) (3F/3M, age 30.5 ± 16 years, range = 19–55). These noise levels (55 dB SPL and 0.5 mA) were selected as our previous work showed they near optimal in terms of inducing SR for a majority of subjects we had tested (Sherman et al., 2023a,b). It should be noted that for within modality perception improvement (i.e., auditory noise to improve auditory signal detection) that low signal-to-noise ratios are required (Zeng et al., 2000; Moss et al., 2004). However, cross-modal perception improvement and cognitive enhancement paradigms using noise have required high levels of noise (~55–70 dB SPL) (Manan et al., 2012; Othman et al., 2019). For AWN stimulation, broadband AWN (20 Hz–20,000 Hz) was administered to subjects through ear buds (Essential Earphones HD) and a Samsung Tablet A; the auditory profiles were developed and calibrated by Creare LLC (Hanover, NH). For nGVS stimulation, broadband, unipolar, zero-mean white noise (0 Hz–100,000 Hz) was bilaterally administered to subject mastoids through the Galvanic Vestibular Oscillating Stimulator (model 0810, Soterix Medical, Woodbridge, NJ) using electrodes with a contact area of 2 cm^2^ (Voros et al., 2021). Traditionally, nGVS frequency profiles have used 640 Hz or less as the high frequency cutoff (Inukai et al., 2018); however, our group has found success in inducing cross-modal perception improvements using this profile (Voros et al., 2021). In the sham treatment, no sensory noise was administered, but subjects were equipped with electrodes and earbuds. Subjects in all treatment groups were fit with AWN and nGVS hardware, independent of whether they actually received sensory noise stimulation.

In their initial visit, subjects watched an 8 minute tutorial video to orient them to the lunar rover simulation environment (see below for details on rover simulation). They then completed one run of the simulation to become familiar with the motives and controls of the simulation. This was done under the guidance of a test operator, which helped explain the rules of the simulation, while avoiding telling them how to do well in the simulation or giving them an opportunity to practice and thus reduce our ability to assess learning.

**Figure 1:**
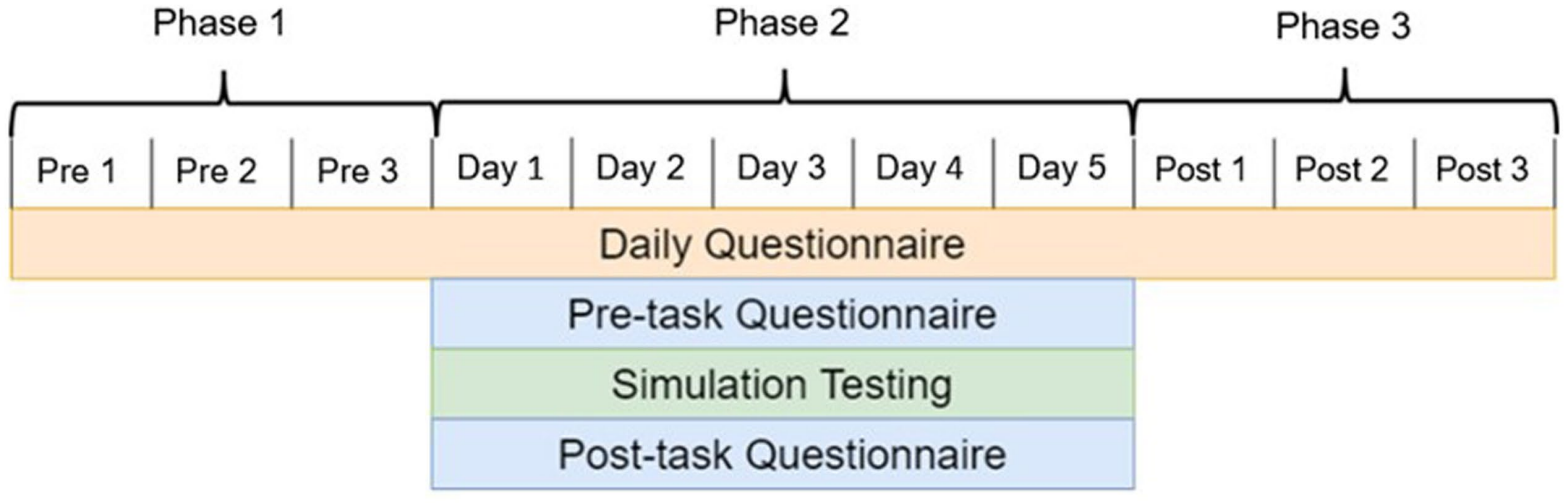
Experimental timeline split up into three distinct testing phases. The first phase is a three-day behavioral health metric collection period to serve as a baseline. The second phase consists of five days of simulation testing under treatment stimulation. The third phase is a three-day collection period to observe after-effects of treatment stimulation.

For this time-longitudinal experiment, all subjects followed a strict, 11 day timeline, which is displayed in Figure 1. This 11 day timeline is comprised of three phases. Phase 1 served as a three-day baseline assessment of behavioral health prior to any treatment stimulation being applied. Phase 2 was the five-day simulation testing period where subjects completed lunar rover simulations under the influence of one of the stimulation treatments. Phase 3 was a post treatment stimulation assessment that allowed us to identify aftereffects as a result of repetitive treatment stimulation. Across all 11 days, an online daily questionnaire was completed in the morning. In addition, subjects completed a questionnaire before and after simulation testing in phase 2.

Metrics of mood, stress, and sleep were collected over the course of the experiment to analyze behavioral health effects of repeated noise exposure. An additional metric of SR acceptability was collected to analyze the acceptability of the noise treatments (Supplementary Data Sheet 1). This was administered after each day’s test session, and then once at the end of the five-day simulation testing period in reference to overall final acceptability. This day 5 questionnaire asked the same questions as the first 4 days but asked subjects to report their overall opinion across the 5 days rather than that specific day. Table 1 details the assessment tools that were used to assess these behavioral health effects.

**Table 1:** Experimental questionnaires and their associated assessment tools.

Questionnaire	Metric	Assessment tool	Reference
Daily Questionnaire	Stress	Stress in General (SIG)	Fuller et al. (2003)
	Mood	Profile of Mood States – Short Form (POMS-SF)	Terry et al. (2003)
	Sleep	Consensus Sleep Diary (CSD)	Carney et al. (2012)
Pre-task Questionnaire	Stress	Short Stress State Questionnaire (SSSQ)	Helton (2004)
Post-task Questionnaire	Stress	SSSQ	Helton (2004)
	Mood	POMS-SF	Terry et al. (2003)
	Acceptability	SR Acceptability Questionnaire (SRAQ)	In house

### Lunar rover simulation

The Lunar Rover Simulation (LRS) is a complex, operational task that allows for the assessment of learning through daily use. Subjects interact with the LRS using an HTC Vive Pro head mounted display to view the lunar landscape and a Logitech X-52 Pro HOTAS joystick to operate the rover. The LRS environment was developed and modified in Unity pulling existing assets from another lunar rover simulation our broader group developed (McGuire et al., 2018). This simulation was designed to feature aspects of the lunar environment, such as; realistic terrain, varied crater sizes, representative lunar lighting and lunar gravity. The LRS is comprised of two operational sub-tasks: path optimization and object identification.

For the path optimization subtask, subjects navigated their rover to several waypoint target destinations along the lunar surface with the goal of minimizing their battery consumption between each waypoint. Power consumption used the specific energy rate equations defined by Carr (2001), where the total power was the sum of the power consumed while moving on a level plane or slope, and a constant power drain (Eq. (1)).


(1) $$W_{\text{Total}}=W_{\text{level}}+W_{\text{slope}}+P_{e}$$

**Figure 2:**
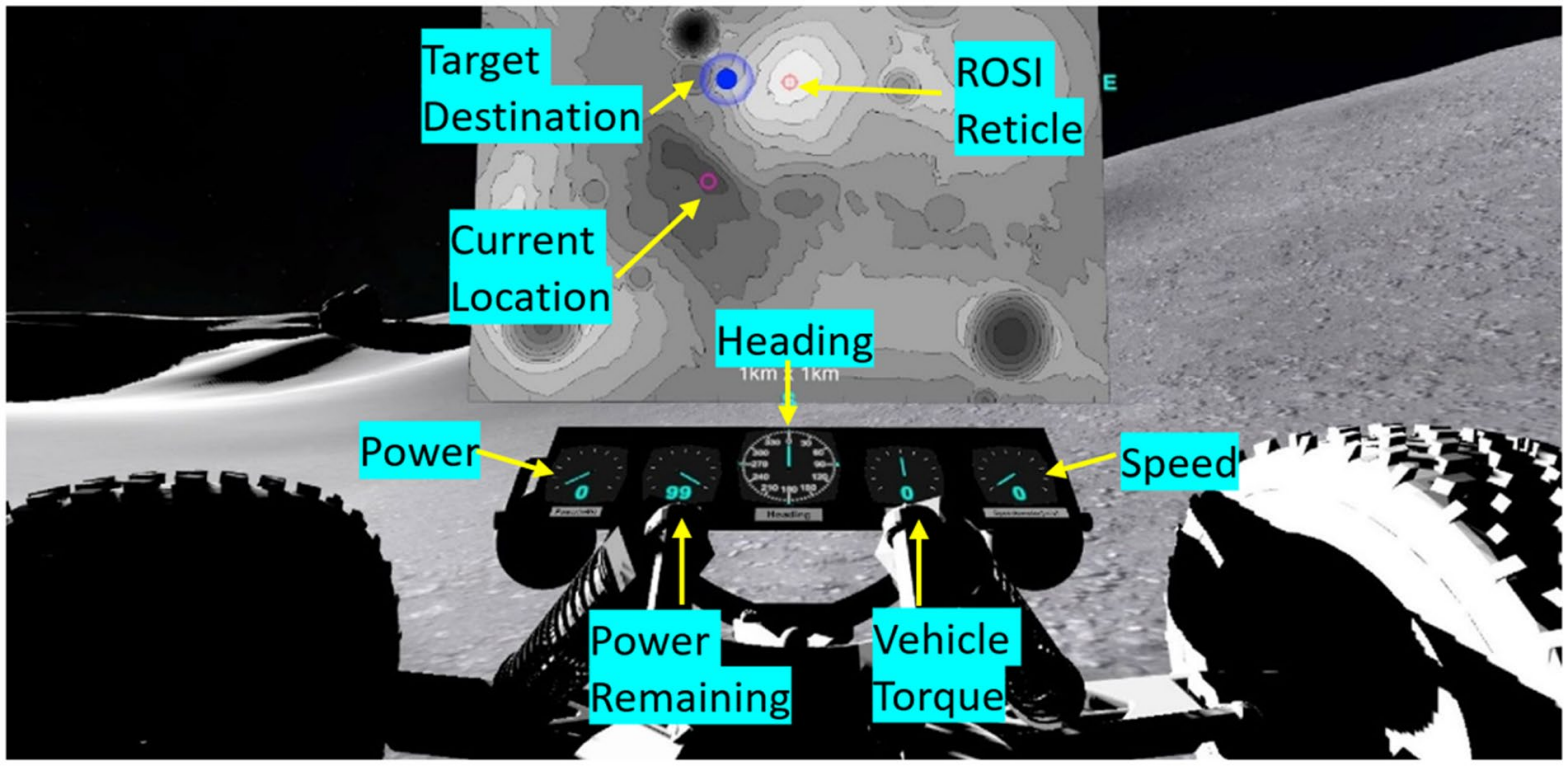
Rover display tools that were provided to the subject. Subjects could pull up a geo-fixed 2-D topographical map of the landscape (shown in this example, top middle), where their location was represented as a magenta circle and the destination waypoint was a blue animated marker. Additional dashboard elements that were continuously available to subjects included current power consumption, battery power remaining, torque, vehicle speed, and body-centered heading. A red reticle could be moved along the topographical map for subjects to tag Rock of Scientific Interest (ROSI) locations.

Battery power consumption was a factor of speed, slope angle, and time (either driving or remaining still, due to constant power drain) across the total distance traveled. Subjects had to learn to weigh each of these factors in optimizing their navigation paths. Subjects were given a variety of tools to help them plan a power-efficient traverse. The first was a 2D topographical contour map of the lunar environment which displayed the waypoint they were navigating to using a blue animated marker with their current location being represented with a magenta circle (see Figure 2, top middle). Subjects could use this tool to plan their traverse trajectory through terrain to optimize power consumption, but they could not move the vehicle while the map was open. Subjects had autonomy on when they looked at and put away the map (selected by button press on the joystick). Subjects were also given a vehicle dashboard which displayed active state information on current power consumption, battery remaining, torque, vehicle speed, and body-centered heading. Figure 2 shows the map and dashboard presented to subjects.

Along with the animated blue marker on the 2D map, subjects knew they had reached their target destination via a stationary 3D robotic rover on the lunar terrain. Once subjects reached their target destination, a new waypoint was presented on the map and their battery was recharged. Each simulation had five waypoints which created a loop; therefore, subjects end where they started. Their starting position in the loop was randomized, but all subjects experienced the same waypoint target destinations. If subjects placed their rover in an unrecoverable position (e.g., overturned in the bottom of a steep crater) or they ran out of battery, the current waypoint trial was considered an “incomplete” and they were teleported to their current target waypoint, which prompted the next traverse.

**Figure 3:**
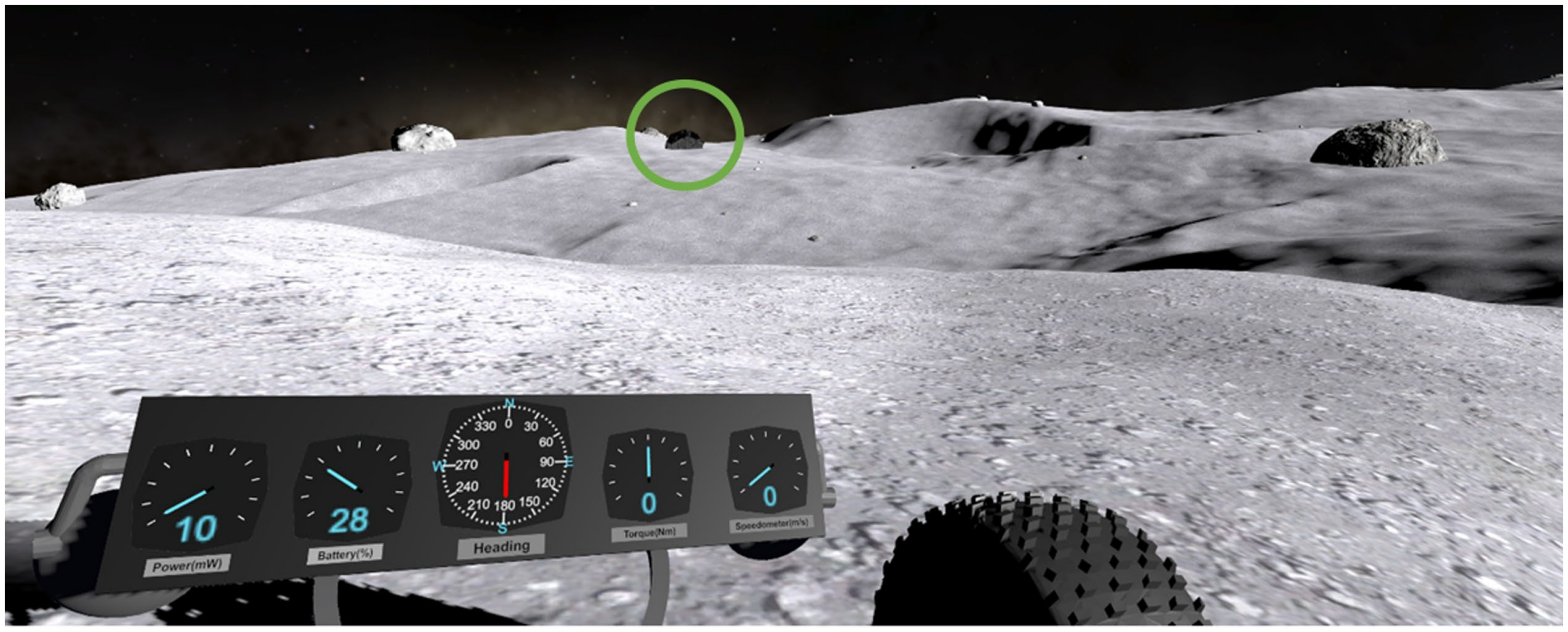
Subject point of view in the object identification task. Upon finding a black ROSI (circled green for the reader), subjects would direct the 2-D map’s red crosshair on their perceived ROSI location and confirm or “tag” its location.

For the object identification subtask, subjects must tag Rocks of Scientific Interest (ROSIs). Several rocks littered the lunar landscape, some of these rocks blended in with the environment and were ubiquitous (dummies); however, the ROSIs were black in color and contrasted the landscape (Figure 3). Subjects were instructed to be vigilant and tag only ROSIs (and not dummy rocks) during their traverses. Subjects tagged ROSI locations via the 2D contour map, moving the red reticle to their perceived ROSI location and laying down a marker (Figure 2). All rock locations were randomized *a priori* and placed in the terrain during development, ensuring all subjects experienced the same rock placements. Based on map design, subjects were expected to see 2–3 ROSIs for each waypoint trial. A unique lunar terrain was given for each test day, such that strategies, skills, and techniques could be learned, but the exact layouts of the lunar terrain and map were not transferrable. The time to complete the LRS depended on the subjects and their strategy; on average, subjects completed the LRS in 20 minutes per session. Subjects received treatment stimulation over the course of completing the LRS, with stimulation not being applied before or after LRS testing.

**Figure 4:**
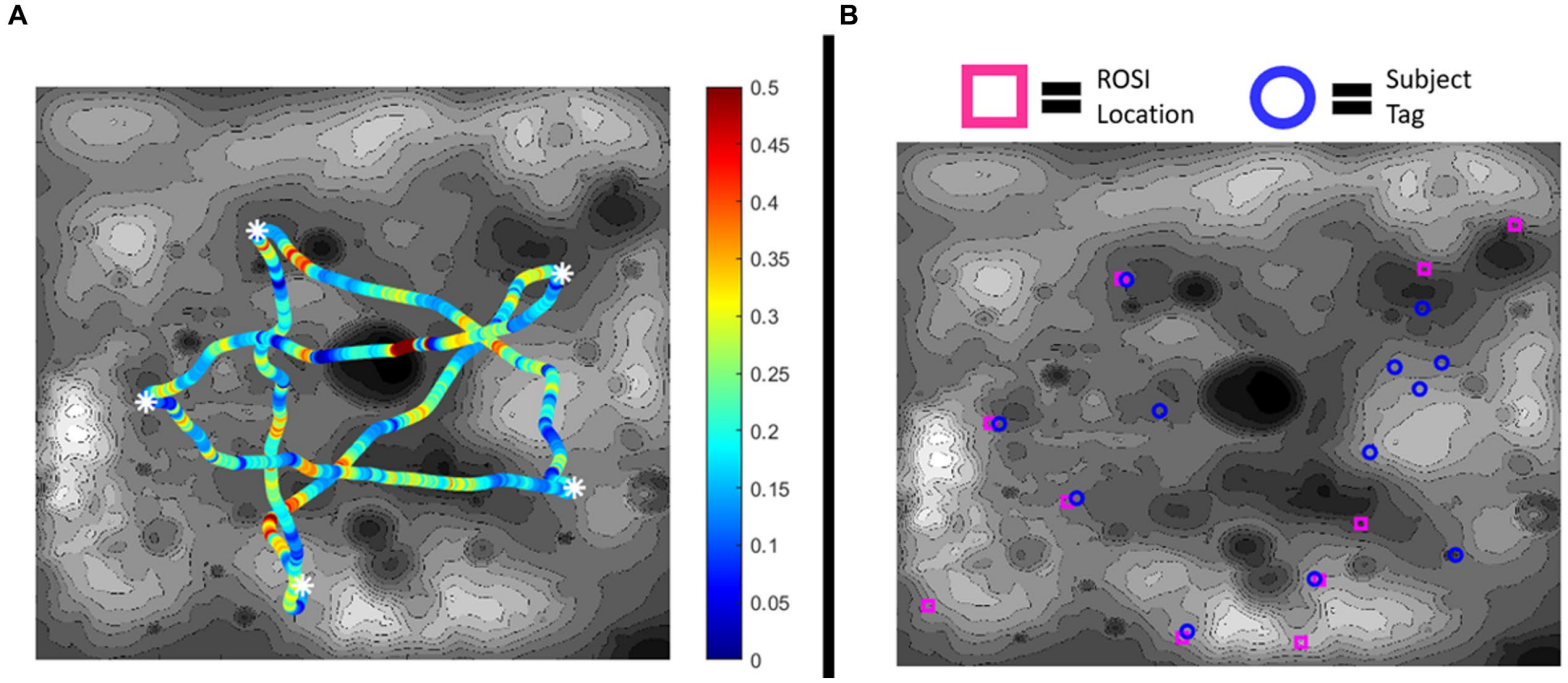
**(A)** Example of subject battery consumption along traverses in the path optimization subtask. White asterisks represent the waypoint locations. Low battery consumption (Watts) is represented by blue in the spectrum and high battery consumption is represented by red. **(B)** Example of subject object identification. Actual ROSI locations are marked in magenta, while subject reported rock tags are marked in blue. In this example, five of the rock tags correspond to ROSI locations but seven do not (i.e., incorrectly identifying a dummy rock as being a ROSI), while five ROSIs were left untagged.

Figure 4 visualizes an example result of one subject’s operational performance in a map. Figure 4A represents their performance in the path optimization subtask with power consumption along a traverse being color coded. Figure 4B visualizes their rock tag placements in contrast to the actual ROSI locations.

### Analysis

In summary, analysis approaches differed between operational learning performance and behavioral health effects. Learning was assessed as changes in operational performance over the five-day testing phase (Phase 2), whereas, group behavioral health differences were considered across all phases (Figure 1). Several Analysis of Variance (ANOVA) models were applied to analyze the effect of the noise treatments. Assumptions for residual normality in learning were calculated using the Anderson-Darling test (*p* > 0.35) to ensure that parametric statistics were appropriate. Additionally, Bartlett’s test was used to test variance homogeneity between groups in the behavioral health analysis (*p* > 0.3). If the omnibus F-test results from the ANOVAs were significant, Tukey HSD multiple pairwise comparisons were used to identify which treatments were different from one another.

### Learning analysis

Performance scores in a traverse for the path optimization subtask was defined as the “battery needed” to reach the desired waypoint. If a subject reached the waypoint, this was simply the amount of battery consumed since the start of the traverse. If a subject failed to reach the waypoint as a result of an “incomplete,” the battery needed term was calculated as 100 (the total amount of battery allotted) plus the battery that would be used to traverse the distance remaining to waypoint (at full speed with no sloped terrain). While this additional term may not accurately reflect subject driving behavior, it prevents the performance ceiling effects of incomplete traverses (if they all remain at 100) and weighs incomplete traverses that made it closer to the waypoint target more favorably than those further away. The performance (P) used in our statistical model (Eq. (3)) for each day was the summation of the five “battery needed” traverse scores for the single LRS divided by five (i.e., a value of 100 corresponded to the full battery consumed on each of the 5 traverses, while lower values corresponded to better performance since less battery was consumed, and higher values were the result of some incomplete traverses and thus worse performance).

Performance scores in the object identification subtask relied on the total number of correct ROSI identifications on a given map. This was done by identifying which rock (ROSI or dummy) was closest to the user’s tagged location. Correct identifications (c) were selected if a ROSI was the closest rock to this tagged location and incorrect identifications (i) were marked if a dummy rock was closest. Identification scores (${\text{ID}}_{P})$ for a given map were calculated using Eq. (2) to reward correct tags and penalize incorrect tags. This was standardized by dividing the result by 10, as there were only 10 ROSIs in each map. The performance (P) used in our statistical model (Eq. (3)) for each day was this ${\text{ID}}_{P}$ score.


(2) $${\text{ID}}_{P}=\left(c-i\right)/10$$

To observe between subject differences in path optimization or object identification performance (P), a mixed effects model was utilized with day (D) was a continuous covariate. Map (M, as a categorical variable) was also included as a covariate to capture variations simply due to the difficulty of the map. A fixed effect of noise treatment (NT) was included to evaluate whether treatment influenced operational performance independent of learning. Finally, to assess differences in learning between the four groups, an interaction term between treatment and day was included in the model. The interaction accounts for the slope of performance improvement between treatment groups. The final analytical model for the two operational subtasks is given in Eq. (3).


(3) P~NT+M+D+NT∗D

### Behavioral health analysis

Following the guidance of previous studies that validated the assessment tools from Table 1, quantifiable metrics of behavioral health were defined. Stress and mood were considered when assessing immediate behavioral health effects from noise stimulation. For mood, the total mood disturbance (TMD) metric was calculated by adding the raw score responses of tension, depression, anger, fatigue, and confusion and then subtracting the vigor score (Terry et al., 2003); thus, lower scores indicate more stable mood profiles. Stress metrics of engagement, distress, and worry were calculated by adding the raw score response of the questions associated with that metric (Helton, 2004). We cared about deviations in behavioral health after task completion and stimulation; thus, the final metric scores being statistically assessed were the behavioral health metric post testing minus the behavioral health metric pretesting.

Following our objectives, we wanted to know whether repeated noise exposure over time impacted behavioral health (B); thus, behavioral health metrics were observed across the 5-day simulation testing period. A two-way ANOVA was used to observe between differences in mood and stress. For this, categorical variables of treatment, day, and their interaction were used, allowing us to understand changes in behavioral health state. The final analytical model used for these behavioral health states is given in Eq. (4).


(4) B~NT+D+NT∗D

Expanding on this, we wanted to understand longitudinal behavioral health effects, seeing whether repetitive noise administration affected behavioral health in the long term (during or afterwards). With respect to the daily questionnaires which loaded questions related to mood, strain, and sleep, we completed two-way ANOVAs to assess differences between the three testing phases (Figure 1). This allowed us to understand how the treatments impacted behavioral health during and after the stimulation testing period, which could suggest long-term after-effects. The categorical testing phases (TP) assessed were the 3 days prior to testing, the 5 days of testing, and the 3 days after testing. Categorical variables of treatment, test phase, and their interaction were used, allowing us to understand changes in behavioral health state across the three test phases. Longitudinal behavioral health measures were standardized by subtracting the average in each subject’s baseline measures. The means were calculated for each test phase in each subject and compared to reduce the weighting of the second test phase (as there were separate measures in this period compared to the three measures collected in the other periods). The final analytical model used for these behavioral health states is given in Eq. (5).


(5) B~NT+TP+NT∗TP

Finally, based on the structure of the acceptability questionnaire (Supplementary Data Sheet 1), raw scores at the conclusion of stimulation were assessed in each of the six questions. A one-way ANOVA was used to understand general acceptability of the stimulation treatments.

## Results

The results are presented in terms of first the operational performance improvement (i.e., learning effects) and second the behavioral health impacts.

### Learning results

**Figure 5:**
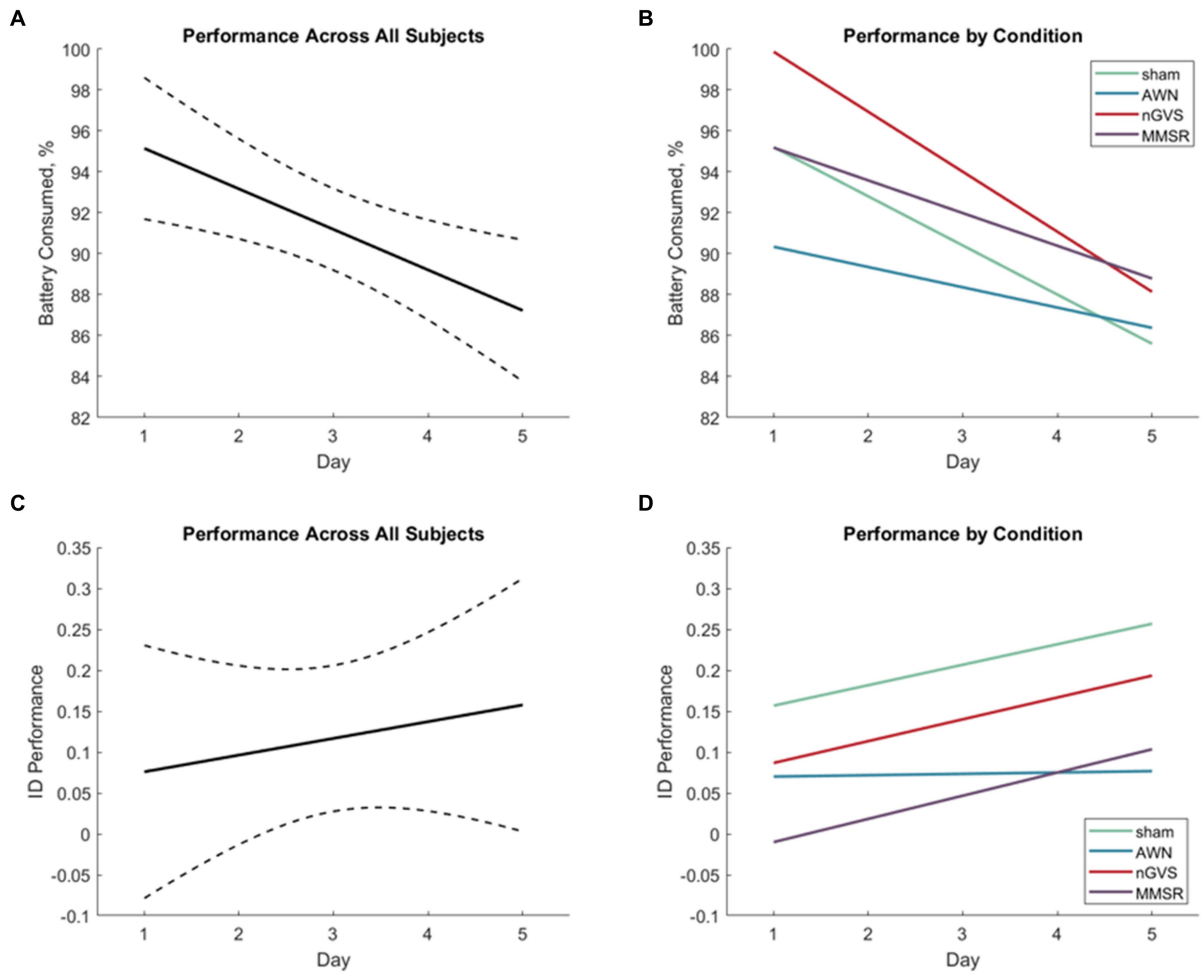
**(A)** Linear regression of path optimization performance improvement across the five test sessions for all subjects. Dashed lines indicate the 95% confidence interval of the modeled fit. **(B)** Linear regressions of path optimization performance improvement across the five test sessions for each treatment group. **(C)** Linear regression of object identification performance improvement across the five test sessions for all subjects. Dashed lines indicate the 95% confidence interval of the modeled fit. **(D)** Linear regressions of object identification performance improvement across the five test sessions for each treatment group.

These results explore learning through changes in operational performance over time in the path optimization and object identification subtasks. Overall needed battery consumption was the metric of performance in path optimization and ID performance (Eq. (2)) was the metric for rock identification. Figure 5 shows the rates of operational performance change in these metrics for all subjects (Figures 5A,C) and by treatment group (Figures 5B,D). Table 2 shows the results of the statistical tests produced by Eq. (3). Significant effects of day were identified in each learning subtask (indicating learning across all subjects); however, the interaction effects were not significant (indicating no difference in learning between treatment groups).

**Table 2:** Mixed effect model results for operational learning performance, for each operational sub-task of path optimization and object identification.

Factor	Path optimization			Object identification		
	F (dof)	*p*-value	$\eta_{p}^{2}$	F (dof)	*p*-value	$\eta_{p}^{2}$
Noise Treatment	1.74 (3, 108)	0.16	0.046	0.23 (3, 108)	0.87	0.006
Map	0.94 (4, 108)	0.44	0.034	5.98 (4, 108)	<0.005*	0.181
Day	13.98 (1, 108)	<0.005*	0.115	3.86 (1, 108)	0.05*	0.035
Noise Treatment*Day	0.66 (3, 108)	0.58	0.018	0.15 (3, 108)	0.93	0.004

Asterisks represent metrics that met a statistical significance below *α* = 0.05.

For the path optimization task, since map appeared to not be a statistically significant factor in the model, we followed up with a simplified model without the map factor to add extra statistical power to the other factors considered. However, this removal did not induce significance in the other factors (*p* > 0.19). This was not applied to the object identification data as map was a significant factor. This technique was not applied to the behavioral health data as it was important to understand the factor effects of treatment and day to identify time longitudinal changes in behavioral health. Contrary to our hypothesis, we did not observe improvements in learning for the noise stimulation treatments, for either path optimization or objective identification performance. Further visualizations of these results, separated by group with representative variance, can be found in Supplementary Image 1.

### Behavioral health results

#### Immediate behavioral health results

We explored the differences in mood and stress prior to and after completing the task with treatment stimulation. Table 3 show the statistical test results given by Eq. (4). Visualizations of these results can be found in Supplementary Image 2.

**Table 3:** Two-way ANOVA results for behavioral health effects following stimulation, split between mood and the three metrics of stress.

Factor	F (dof)	*p*-value	$\eta_{p}^{2}$	F (dof)	*p*-value	$\eta_{p}^{2}$
	TMD			Engagement		
Noise Treatment	2.23 (3, 100)	0.09	0.063	1.6 (3, 100)	0.19	0.046
Day	0.4 (4, 100)	0.81	0.016	0.84 (4, 100)	0.5	0.033
Noise Treatment*Day	1.22 (12, 100)	0.28	0.128	2.1 (12, 100)	0.02*	0.202
	Distress			Worry		
Noise Treatment	1 (3, 100)	0.39	0.029	0.5 (3, 100)	0.68	0.015
Day	0.73 (4, 100)	0.57	0.029	0.5 (4, 100)	0.74	0.02
Noise Treatment*Day	1.42 (12, 100)	0.17	0.145	1.42 (12, 100)	0.17	0.146

Asterisks represent metrics that met a statistical significance below 0.05.

We identified a significant interaction between noise treatment and day for the stress metric of engagement (Supplementary Image 2); however, no other factors were significant. Contrary to our hypothesis, it appears there are no strongly influential effects of stimulation on immediate behavioral health.

#### Longitudinal behavioral health results

We wanted to observe differences in behavioral health between the three time periods; pre-testing baseline, testing with stimulation, and post-testing aftereffects. Table 4 show the statistical test results given by Eq. (5). Visualizations of these results can be found in Supplementary Image 3.

**Table 4:** Two-way ANOVA results for longitudinal behavioral health effects between treatments, split between mood, strain (three metrics), and sleep (three metrics).

Factor	F (dof)	*p*-value	$\eta_{p}^{2}$	F (dof)	*p*-value	$\eta_{p}^{2}$
	TMD			Relaxed and Calm		
Noise Treatment	0.02 (3, 60)	0.99	0.001	1.51 (3, 60)	0.22	0.07
Period	0 (2, 60)	0.99	<0.001	0.49 (2, 60)	0.61	0.016
Noise Treatment*Period	0.19 (6, 60)	0.98	0.019	0.48 (6, 60)	0.82	0.046
	Comfort and Smooth			Pushed and Stressed		
Noise Treatment	2.65 (3, 60)	0.06	0.117	1.09 (3, 60)	0.36	0.052
Period	2.18 (2, 60)	0.12	0.068	0.2 (2, 60)	0.82	0.007
Noise Treatment*Period	0.88 (6, 60)	0.51	0.081	0.77 (6, 60)	0.6	0.071
	Total Sleep			Sleep Quality		
Noise Treatment	0.89 (3, 60)	0.45	0.042	1.38 (3, 60)	0.26	0.064
Period	0.21 (2, 60)	0.81	0.007	0.01 (2, 60)	0.99	<0.001
Noise Treatment*Period	0.52 (6, 60)	0.79	0.049	0.42 (6, 60)	0.86	0.041
	Feeling Refreshed					
Noise Treatment	5.16 (3, 60)	<0.005*	0.205			
Period	1.13 (2, 60)	0.33	0.036			
Noise Treatment*Period	1.34 (6, 60)	0.24	0.118			

Asterisks represent metrics that met a statistical significance below *α* = 0.05.

We identified a significant effect of noise treatment for the sleep metric of “feeling refreshed”; however, no other factors were significant. Multiple comparisons showed that the AWN treatment group was significantly more refreshed than the sham group (*p* < 0.005). Contrary to our hypothesis, it appears there are no strongly influential effects of stimulation on longitudinal behavioral health since only this difference was identified.

### Acceptability results

We developed an acceptability questionnaire (Supplementary Data Sheet 1) to assess differences in stimulation acceptability between treatment groups. Table 5 show the statistical test results for the resulting one-way ANOVAs. Visualizations of these results can be found in Supplementary Image 4.

**Table 5:** One-way ANOVA results for the six metrics of treatment acceptability.

Metric	F (dof)	*p*-value	$\eta_{p}^{2}$
Felt the equipment did not inhibit performance	2.43 (3,116)	0.07	0.059
Felt that the AWN was comfortable	5.55 (3,116)	<0.005*	0.126
Felt that the nGVS was comfortable	0.93 (3,116)	0.43	0.023
Felt that they were able to maintain focus	2.31 (3,116)	0.08	0.056
Found the treatment stimulation was distracting	5.07 (3,116)	<0.005*	0.116
Found the treatment stimulation fatigued them	2.52 (3,116)	0.06	0.061

Asterisks represent metrics that met a statistical significance below *α* = 0.05.

**Figure 6:**
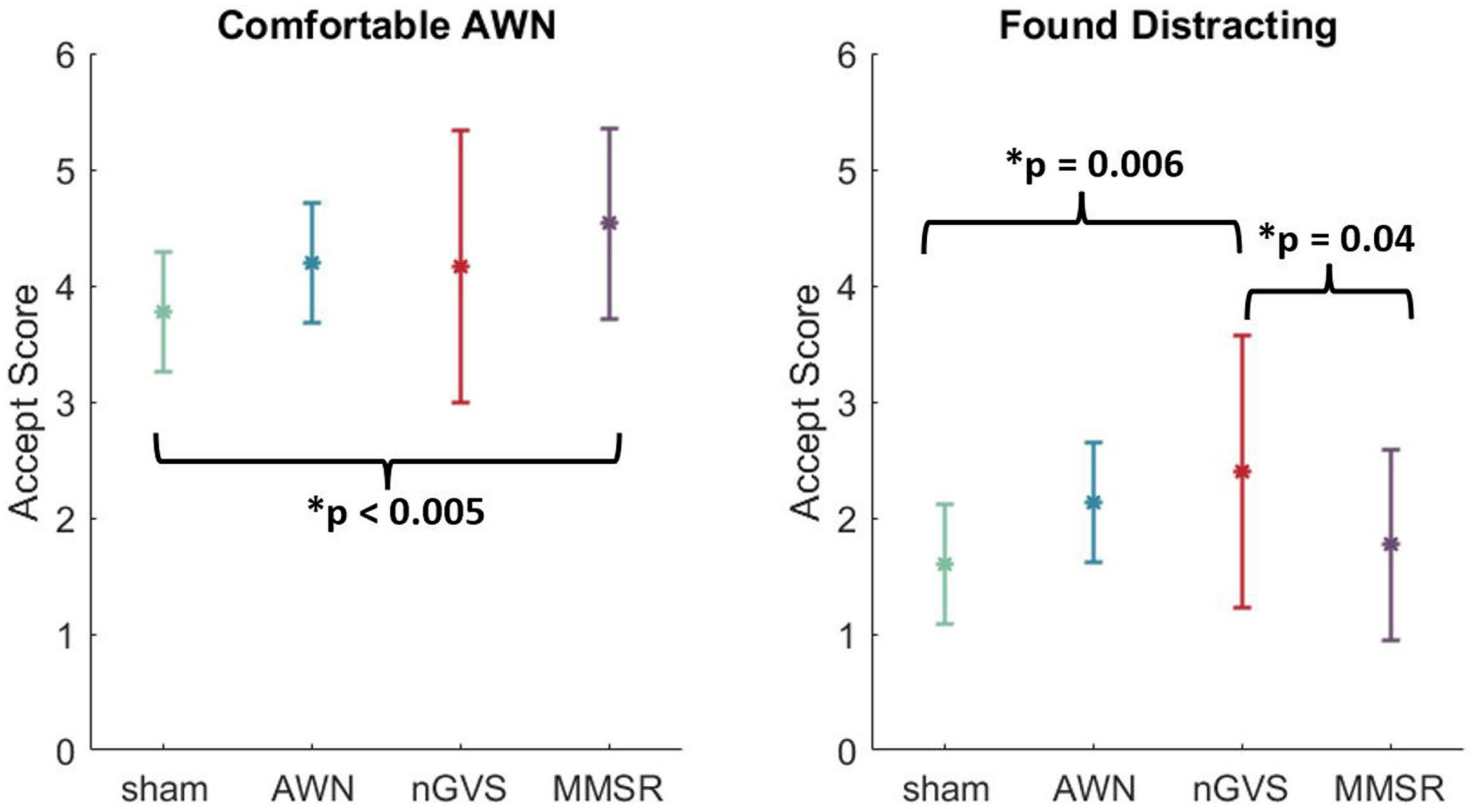
Multiple comparisons in the two significantly different acceptability metrics. Error bars indicate the standard deviation of the data point. Brackets indicate the treatments that were identified to be different from one another.

We identified a significant effect of noise treatment for the acceptability metrics of “AWN was comfortable” and “stimulation was distracting.” No other acceptability questionnaire metrics were significant. For the first significant metric, a multiple comparison analysis showed that the MMSR treatment group believed the AWN stimulation was significantly more comfortable than the sham group (*p* < 0.005). For the second significant metric, a multiple comparison analysis showed that the nGVS treatment group believed the nGVS stimulation was significantly more distracting than the sham group (*p* = 0.006) and the MMSR group (*p* = 0.04). These results are visualized in Figure 6. Independent of these two metrics, it appears that sensory noise stimulation is generally deemed to be acceptable between the treatment groups.

## Discussion

This research investigated for the first time the effects of repetitive sensory noise stimulation on operational learning, as well as its long-term effects on behavioral health. This was done by having subjects complete a lunar rover simulation once daily, for 5 days, under treatment stimulation. While we found subjects broadly performed better in the operational task across days, there were no differences in the rate of task improvement between groups (i.e. no differences in learning). Prior, post, and during this period, subjective questionnaires related to behavioral health metrics of mood, stress, and sleep were collected. We found no significant differences in behavioral health or acceptability between treatment groups except in a few specific metrics.

Previous sensory noise literature related to memory found that nGVS can improve spatial memory and AWN can improve auditory working memory (Hilliard et al., 2019; Othman et al., 2019); however, the study presented here was not able to find significance. Focusing on the study conducted by Hilliard et al. (2019), subjects completed a within-subject cross-over design with a virtual spatial memory task under the influence of nGVS stimulation (tailored to 80% of their sensation threshold) in one of two sessions that were separated by 2 weeks. During testing subjects were tasked to explore a virtual arena, learn the location of objects, and then mark their location when they were removed. They completed three of these runs. This study found improved accuracy of object location across the learning run. It should be noted that Hilliard et al. (2019) tested more subjects than ours, but we want to call into question the use of nGVS for declarative vs. non-declarative memory formation, where procedural memory tasks are non-declarative in nature (Brem et al., 2013). The task completed in this study was procedural, whereas Hilliard et al. (2019) object location task can be argued as declarative. It is possible that the memory formation paradigm that SR targets is semantic and declarative in nature, but further investigations into procedural memory formation paradigms are needed.

The closest procedural paradigm similar to the experiment presented here was found for alternative forms of neuromodulation which had observed that neural stimulation from tDCS applied to the right dorsolateral prefrontal cortex can lead to improved learning rates in complex operational tasks as opposed to receiving no stimulation (Choe et al., 2016). Choe et al. (2016) investigated operational learning in an aviation landing simulation across 4 days. In this task subjects aimed to replicate a landing that was similar to an autopilot demonstration that was presented to them before their simulation began. To do this, subjects used instrumentation cues during the autopilot demonstration to guide their landing. For the simulation paradigm we investigated, this type of replication scenario was not represented, as our task was a self-guided learning paradigm. Results compiled across all subjects suggest that performance in the operational task significantly improved across all subjects over time, but there was no difference between stimulation treatments and sham. This indicates the lunar rover simulation paradigm could still capture effects of learning. This could suggest that our results imply that additive sensory noise is not an appropriate neuromodulation technique for learning enhancement within this group or that the noise treatment produces sufficiently low effect sizes that this experiment is not sensitive to. Yet, it is entirely possible that we are assessing the incorrect learning task mode to demonstrate SR improvements as the tDCS study referenced in this paper used a replication paradigm.

While sensory noise may not improve learning within this experimental paradigm for this population, it has been shown that noise can lead to broad performance enhancement within specific use cases. Multiple studies have shown that SR is broadly exhibited in perception, both within and across separate sensory modalities (Lugo et al., 2008; Galvan-Garza, 2018; Voros et al., 2021). This implies that certain attributes of information processing can be enhanced by sensory noise for a broad population. However, when it comes to inducing cognitive enhancements, the results appear to be inter-individually driven. Wilkinson et al. (2008) found that nGVS improves facial recognition recall in healthy, neurotypical participants, but Söderlund et al. (2010) and Chen et al. (2022) found auditory noise improves cognitive processing in only inattentive children, worsening cognition in attentive children. This is furthered by a recent investigation which observed mixed results for AWN and cognition in neurotypical subjects (Awada et al., 2022). Further, research conducted by our lab found no broad cognitive enhancements under sensory noise, but found that subject interactions were significant, with subjects that self-reported being able to work better with background noise showing cognitive enhancement as a result of sensory noise treatment (Sherman et al., 2023a). This implies that applying sensory noise for cognitive processing may only be beneficial for certain individuals. Our study used a novel operationally complex learning paradigm with a neurotypical population, which shows mixed results for noise affecting cognitive influence. Additional investigations are necessary to determine if there are separate paradigms or specific individuals would see learning enhancement due to noise.

However, it is useful to understand whether sensory noise has secondary effects to behavioral health which would undermine the usage of sensory noise in these specific use cases or individuals.

Alternative neuromodulation techniques, specifically tDCS and TMS, have been shown to create immediate, lasting effects on neuronal excitability and long-term behavioral health (Mantovani et al., 2012; Medeiros et al., 2012). A suitable neuromodulation technique for repeated administration would not negatively affect behavioral health, especially on a long-duration space mission. Since sensory noise effects on behavioral health have not been observed in the literature, we aimed to address this gap. Behavioral health questionnaires following stimulation and testing allowed us to assess immediate behavioral impacts. The longitudinal daily collection of behavioral health questionnaires related to mood, strain, and sleep allowed us to assess sensory noise effects on general behavioral health and potential aftereffects. In general, our results do not suggest that sensory noise impacts behavioral health. While you cannot prove a null result, the effect sizes related to most of our metrics suggest that you would need extremely high subject numbers to identify significant differences. For example, a retrospective power analysis for immediate mood changes with a $\eta_{p}^{2}$ = 0.063 (Table 3) showed that 104 subjects are required to identify treatment group differences. With such a small effect size from these validated and sensitive questionnaires, we feel confident that many of the measures related to behavioral health would not result in meaningful impacts from sensory noise stimulation. Inferring from these results and our findings which suggest noise stimulation is generally acceptable (Table 5), we believe that repetitive administration of AWN and nGVS have no effects on behavioral health and is generally acceptable for repeated use in situations and individuals that necessitate its usage.

There are a few limitations to this study that are worth noting. First, previous research conducted for perceptual and cognitive SR have identified that there is a subject and task specific optimal noise level to induce performance enhancement (Ries, 2007; Voros et al., 2021; Sherman et al., 2023a). Since this task is a learning paradigm, there was no efficient way of identifying a subject’s specific optimal noise level. We tried to navigate this problem by choosing the noise levels that were most commonly represented as near optimal in previous cognition investigations completed within this lab (Sherman et al., 2023a,b), which we believed would allow us to produce potential SR effects for a majority of participants in this study. However, it is possible that these levels would not induce SR benefits in terms of improved learning for some or many of our subjects, but that had we applied different levels (or individualized levels) SR benefits may have been observed. Second, our subject number per group (*n* = 6) is relatively low. That being said, we have included effect sizes for future research and meta-analyses. The effect sizes related to operational learning are sufficiently low enough for the interaction terms of our statistical analysis (Table 2) that a few more subjects within each treatment group would probably not yield significant changes. However, this low subject number may explain significant differences in certain ordinal measures. For example, sham subjects believed the AWN stimulation was more uncomfortable than the MMSR group which did receive AWN stimulation (as well as nGVS); the AWN treatment group was not significantly different from either. While is possible that the simultaneous application of nGVS and AWN caused the experience of AWN to be more comfortable, it could suggest that this result is a false positive. Greater sensitivity in the acceptability questionnaire or greater subject numbers may have prevented this result. However, the significant acceptability result of nGVS being more distracting than sham follows with preconceived notions. Finally, it is difficult to speculate on how nGVS effects in an Earth gravity environment translate to similar performance in microgravity. Microgravity induces otolithic deprivation which induces sensory reweighting, especially in the vestibular system (Pathak et al., 2022). While the otoliths are still functional in microgravity and continue to transduce linear acceleration, this could cause an interaction that changes the effects of GVS stimulation in spaceflight. While Lajoie et al. (2021) provide an in-depth review on the potential promise of nGVS for spaceflight human performance and vestibular enhancement, the interaction effects of nGVS with the microgravity-affected vestibular system are still unknown as no spaceflight studies using GVS have occurred to date.

## Conclusion

This investigation evaluates the long-term effects of repetitive sensory noise administration on operational learning and behavioral health. We conclude that applying AWN and nGVS repeatedly does not affect the rate of learning of an operational task for a neurotypical population. Additionally, there appears to be no effects of sensory noise exposure on behavioral health, either immediately or on a longitudinal timescale. We also found that AWN and nGVS stimulation is perceived to be acceptable by subjects. Thus, repeated sensory noise exposure to elicit SR in specific use cases or individuals may be utilized with little side effects.

## Data availability statement

The raw data supporting the conclusions of this article will be made available by the authors, without undue reservation.

## Ethics statement

The studies involving human participants were reviewed and approved by the University of Colorado-Boulder’s Institutional Review Board (protocol #21-0296). The patients/participants provided their written informed consent to participate in this study.

## Author contributions

SS is the first author responsible for analyzing the data, developing the experimental design, and drafting the manuscript. AJ, QL, and MS are co-authors responsible for developing experimental hardware/software and subject testing. DS is a co-author that helped consult on this project and guidance for the original rover simulation environment. TC and AA are co-authors and co-PIs on this research. They helped guide and provide feedback on experimental design, data analysis, and manuscript writing. All authors contributed to the article and approved the submitted version.

## Funding

This study was funded by the Translational Research Institute for Space Health (TRISH) through NASA Cooperative Agreement NNX16AO69A (award number T0402).

## Conflict of interest

The authors declare that the research was conducted in the absence of any commercial or financial relationships that could be construed as a potential conflict of interest.

## Publisher’s note

All claims expressed in this article are solely those of the authors and do not necessarily represent those of their affiliated organizations, or those of the publisher, the editors and the reviewers. Any product that may be evaluated in this article, or claim that may be made by its manufacturer, is not guaranteed or endorsed by the publisher.
