# Supplementary material for: Training augmentation using additive sensory noise in a lunar rover navigation task
Source: Front Neurosci. 2023 Jun 23;17:1180314. doi: 10.3389/fnins.2023.1180314 (PMC10326282; doi:10.3389/fnins.2023.1180314)
Supplement: Supplementary file 1 [file Image_1.pdf]

Appendix B: Operational Learning Results Visualization

Figure AB1 displays the difference between treatment groups by day in the path optimization subtask. Figure AB2 displays the difference between treatment groups by day in the object identification subtask. Error bars represent the standard deviation.

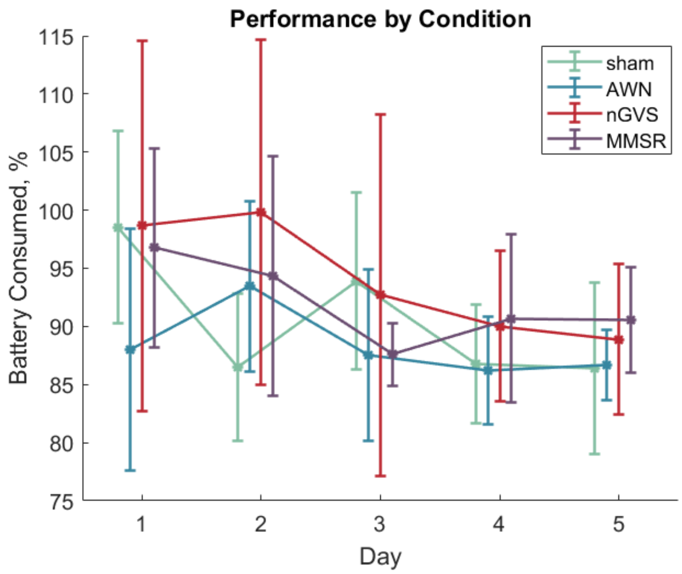

Figure AB1: Needed battery usage by day

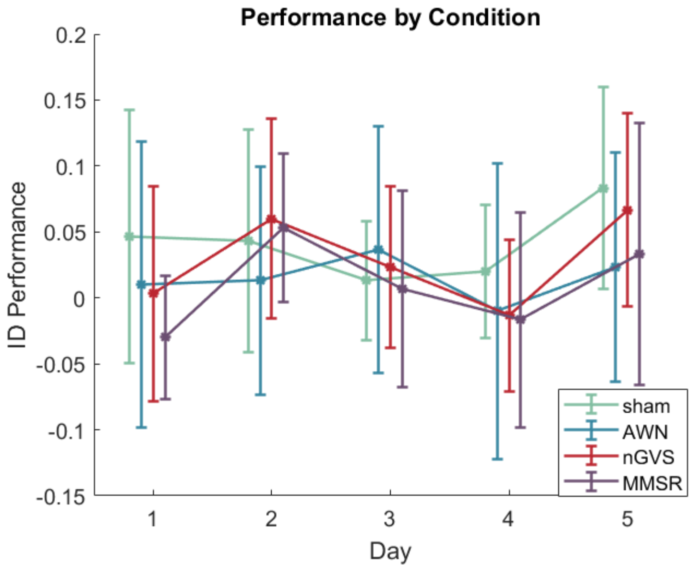

Figure AB2: Rock identification performance by day
