# Supplementary material for: Training augmentation using additive sensory noise in a lunar rover navigation task
Source: Front Neurosci. 2023 Jun 23;17:1180314. doi: 10.3389/fnins.2023.1180314 (PMC10326282; doi:10.3389/fnins.2023.1180314)
Supplement: Supplementary file 2 [file Image_2.pdf]

Appendix C: Immediate Behavioral Effects Visualization

Figure AC1 displays the difference in TMD between treatment groups. Figure AC2 displays the difference in stress metrics (engagement, distress, and worry) identified by Helton (2004) between groups. Error bars represent the standard deviation.

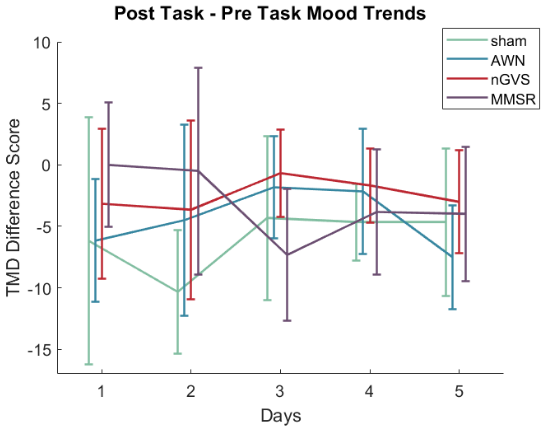

Figure AC1: Immediate mood differences

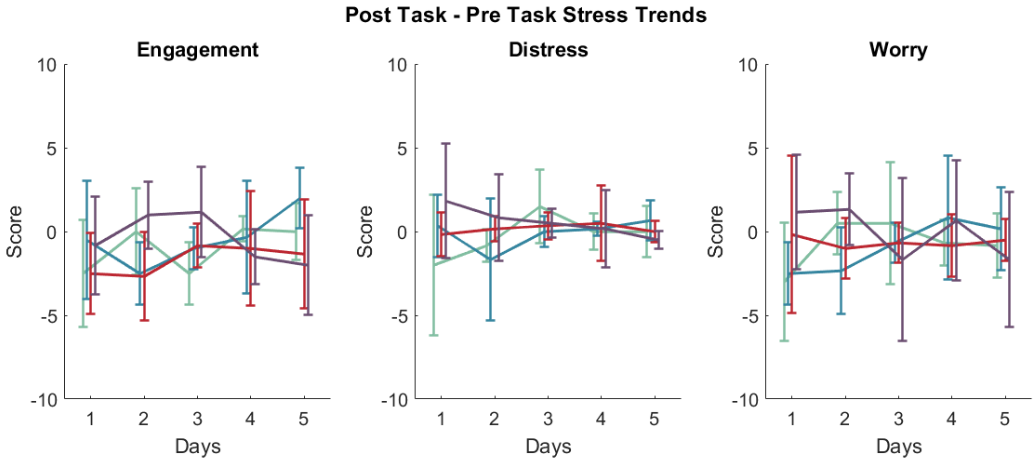

Figure AC2: Immediate stress differences
