# Supplementary material for: Training augmentation using additive sensory noise in a lunar rover navigation task
Source: Front Neurosci. 2023 Jun 23;17:1180314. doi: 10.3389/fnins.2023.1180314 (PMC10326282; doi:10.3389/fnins.2023.1180314)
Supplement: Supplementary file 3 [file Image_3.pdf]

Appendix D: Longitudinal Behavioral Effects Visualization

We assessed measures of mood (Fig. AD1), strain (Fig. AD2), which is comprised of three metrics as defined by Fuller et al. (2003), and sleep (Fig. AD3), which was comprised of three quantitative measures collected by the consensus sleep diary. Error bars represent the standard deviation.

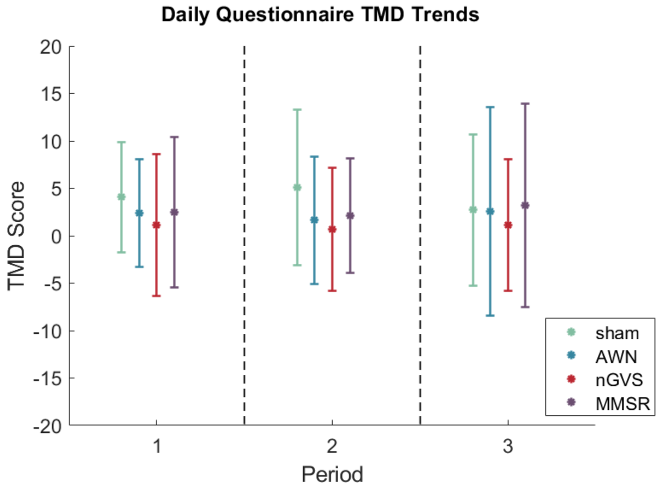

Figure AD1: Longitudinal mood differences

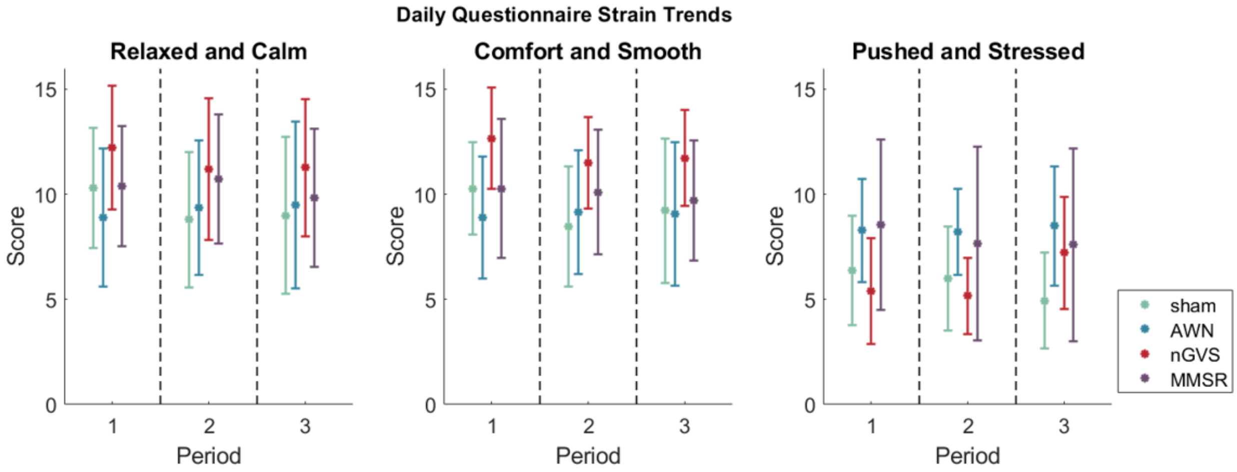

Figure AD2: Longitudinal strain differences

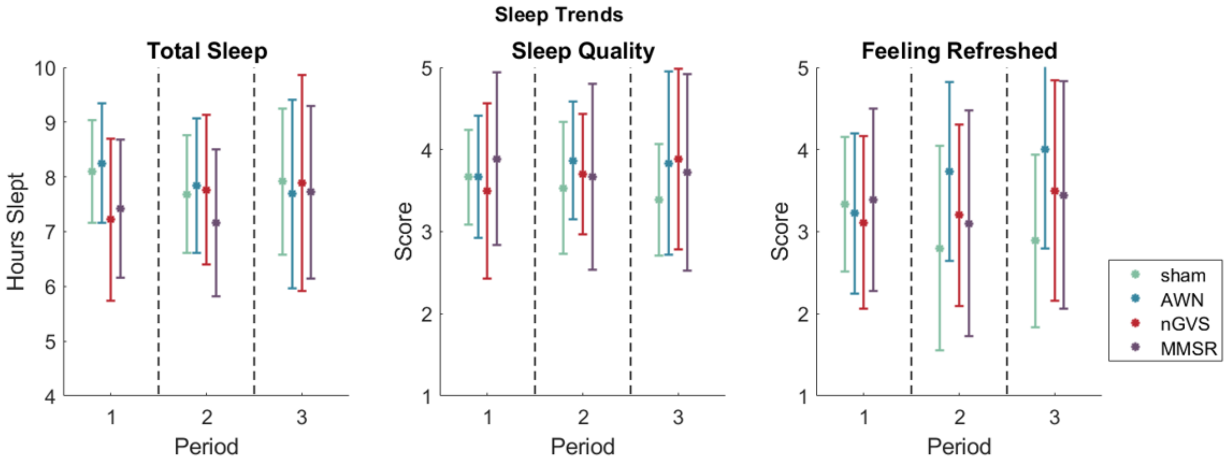

Figure AD3: Longitudinal sleep differences
