# Supplementary material for: Training augmentation using additive sensory noise in a lunar rover navigation task
Source: Front Neurosci. 2023 Jun 23;17:1180314. doi: 10.3389/fnins.2023.1180314 (PMC10326282; doi:10.3389/fnins.2023.1180314)
Supplement: Supplementary file 4 [file Image_4.pdf]

Appendix E: Acceptability Results Visualization

Figure AE displays the difference in our acceptability measures (Appx. A). Error bars represent the standard deviation.

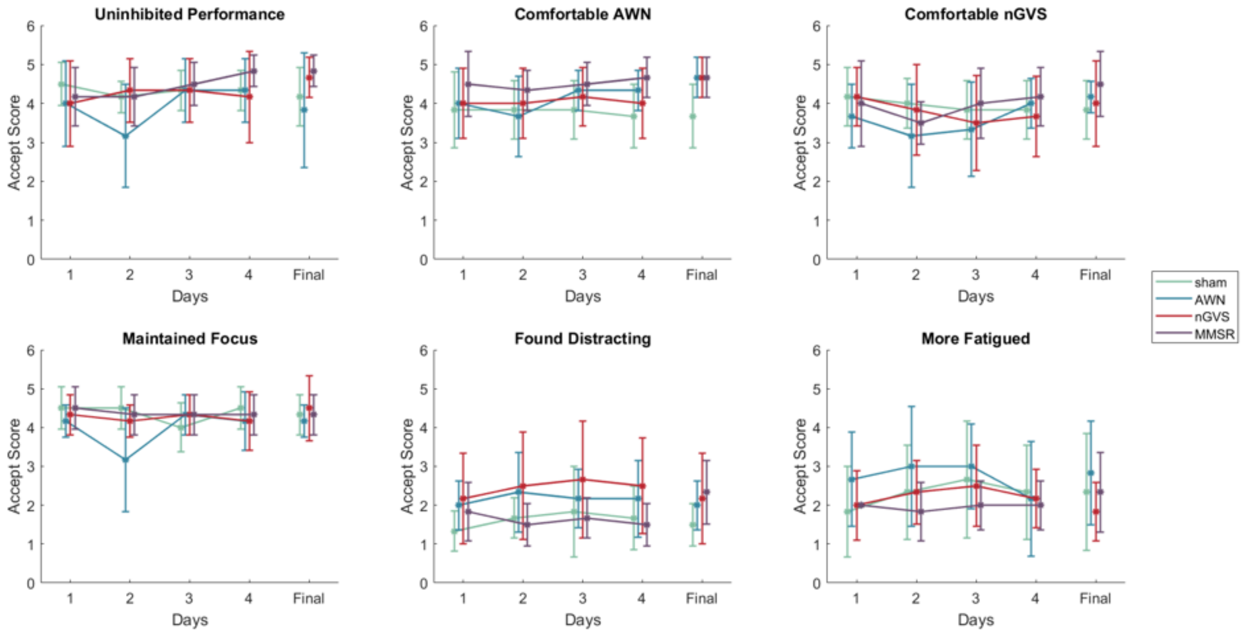

Figure AE: Acceptability differences by day
