# Supplementary material for: Training augmentation using additive sensory noise in a lunar rover navigation task
Source: Front Neurosci. 2023 Jun 23;17:1180314. doi: 10.3389/fnins.2023.1180314 (PMC10326282; doi:10.3389/fnins.2023.1180314)
Supplement: Supplementary file 5 [file Data_Sheet_1.pdf]

Appendix A: Acceptability Questionnaire

The main purpose of this questionnaire is to learn about your experiences with stochastic resonance (SR) as you completed activities. Please base your responses on **today's** experience while wearing the SR delivery equipment and completing tasks.

Please read EACH of the following statements, and indicate the extent to which you agree with each:

**Statement**	**Strongly Disagree**	**Disagree**	**Neutral**	**Agree**	**Strongly Agree**
I was able to perform my tasks uninhibited while wearing the SR devices.					
I found the white noise sound level to be comfortable.					
I found the galvanic-vestibular stimulation comfortable.					
I was able to stay focused while performing tasks.					
I was able to communicate to team members effectively.					
I found the SR stimulation to be distracting.					
I found myself more fatigued than usual at the end of the day.					
